# Right-Wing Populism, Social Identity Theory, and Resistance to Public Health Measures During the COVID-19 Pandemic

**DOI:** 10.3389/ijph.2022.1604812

**Published:** 2022-05-12

**Authors:** Kathleen D. Magnus

**Affiliations:** Institute of Medical Sociology, Center for Psychosocial Medicine, University Medical Center Hamburg-Eppendorf, Hamburg, Germany

**Keywords:** compliance, right-wing populism, COVID-19, mitigation measures, social psychology, superordinate identity, social identity theory

## Abstract

Many western democracies experienced significant resistance to public health measures designed to curb the spread of the COVID-19 virus. Although there were complex reasons for this resistance, right-wing populist forces seem to have played a significant role in fueling it. Studies show a strong correlation between right-wing populist support and resistance to COVID-19-mitigating measures including vaccination, and those who supported these populist movements were more likely to suffer and die from the virus. The question thus arises: why do people support these movements which openly undermine their own health interests? This paper addresses this question from a social-psychological perspective. Specifically, it draws on social identity theory to explain the considerable success of right-wing populism’s radical anti-health agenda and to offer some suggestions about how this negative influence may be countered.

## Introduction

Even before the outbreak of the COVID-19 pandemic, the tendency of right-wing populist movements to oppose measures designed to improve public health was well-established. Opposition to universal health care, climate change mitigation, and international cooperation typically characterizes these agendas [1]. Moreover, studies suggest that right-wing populist parties tend to gain political support when their population’s health declines [2]. Although it is still too soon to predict the full political impact of the COVID-19 pandemic, there is growing evidence that the consequences will lead to an increase in populism, rather than a decline [3].

The recent rise in right-wing forms of populism thus represents a vicious circle difficult to escape. The stronger the hold that extreme right movements obtain on government, the more likely it is that public health will decline and—paradoxically—the more likely support for right-wing populism will increase. Since right-wing populist leaders typically gain power when a population’s health is weak, it is not surprising that they work to defeat public health efforts. What is more difficult to understand is why so many people support populist movements that undermine their own health interests. Propaganda and false information campaigns explain this paradox to a great extent. However, this answer begs the more fundamental question as to why so many people prove susceptible to this kind of misinformation and manipulation. Cognitive biases play a role, but they cannot account for the entire phenomenon [4].

This paper draws on social identity theory, a well-established theory in social psychology, to provide at least a partial explanation. Part one introduces two social identity theory principles that are particularly relevant to the social dynamics of right-wing populism. Part two illustrates how these principles have been operative in right-wing populist attempts to undermine public health campaigns aimed at reducing the spread of the COVID-19 virus. Finally, part three draws on social identity theory to suggest how right-wing populism’s anti-health agenda might be countered. Although it is not possible to do justice to the complexities of the social identity tradition here, its basic principles offer valuable insight into the social and political dynamics that have exacerbated the current public health crisis.

## Part One: Right-Wing Populism Through the Lens of Social Identity Theory

Broadly speaking, populism can be understood as an ideology or movement that claims to support the will of “the pure people” as opposed to “the corrupt elite” [5], p.6. Right-wing populism in particular is tied to additional ideological commitments including an agenda that 1) promotes social division; 2) excludes and denigrates “others,” such as minorities, immigrants, and women; 3) tends toward nationalism, fascism, and authoritarianism; and 4) propagates misinformation and conspiracy theories [6].

Since World War II social theorists and psychologists have struggled to understand the appeal of this kind of destructive ideology. While many sought answers in the study of distinct personality types, others rejected the idea that large-scale social oppression could be merely the result of individual character traits. In the late 1970s Henri Tajfel embarked on a series of experiments designed to identify the social-psychological factors that lead to ingroup bias and outgroup discrimination. Most famously, he and others demonstrated that even when the distinctions between groups are arbitrarily or minimally drawn, people are quick to exhibit ingroup bias: “Mere categorization” is sufficient to elicit ingroup bias [7], p. 281. Although these original experiments used schoolboys as subjects, decades of experiments, some which involved both genders and adults, attest to the robustness of the conclusions [8].

Tajfel and Turner theorized these findings in terms of the individuals’ desire to belong to the winning group and achieve “positive distinctiveness.” People, they claimed, strive for a positive social identity and this identity is “based to a large extent on favorable comparisons that can be made between the in-group and some relevant out-group(s)…” [7] p. 284. A positive self-concept is thus linked to the ability to see the group(s) to which one belongs not simply as good, but as better than some concretely existing other group(s). Unlike Freud, who saw this egoistic drive for superiority as fundamental to group identity formation, these experiments do not suggest that every individual garners his or her self-image this way all the time. They do, however, demonstrate a strong tendency in this direction. Conscious or not, the desire to belong to the “better” group may be far more operative in intergroup dynamics than immediately apparent.

Further experiments showed that what motivated subjects’ allocation decisions more than anything else was the desire to maximize the positive difference between their group and the other group [7]. Indeed—and crucial to the discussion here—the average point distribution indicated a considerable willingness to sacrifice objective gains to ingroup members so long as ingroup members received more than outgroup members. In other words, when subjects had to decide between giving ingroup members the highest net profit and giving them the greatest relative advantage, they preferred the latter option. Additional experiments allowing for direct personal gain demonstrated a similar willingness to sacrifice rewards for the sake of the group’s comparative advantage. In both sets of experiments, the psychological benefits of belonging to the “winning” group played a more determinant role than the prospect of tangible material rewards.

## Part Two: Right-Wing Populism and Resistance to Public Health Measures

Although there may be some difficulties in applying the principles of social identity theory to real-world political identities, most social identity theorists agree that if people are willing to show ingroup favoritism in minimal group situations, their willingness to do so in real-world circumstances, where historical and cultural connections exist, will only be stronger [9]. Tactics employed by right-wing populist leaders illustrate the appeal of these principles particularly well. By promoting an antagonism between “us” and “them,” (“the pure people” vs. “the corrupt elite,” but also the “true” natives vs. immigrants and minorities) [6], they appeal to individuals’ desire to see their own group(s) as “positively distinct” from others. By disparaging outsiders, excluding outgroup members, and scapegoating the weak, they allow their followers to feel “better”. Even when they are not necessarily doing better economically or in terms of physical health, they gain a sense of value simply by marking themselves as belonging to the “in” group. In turn, this group identification reinforces the power of the group leaders.

A similar phenomenon seemed apparent in the right-wing populist response to the pandemic, at least in some countries such as the United States. By appealing to the idea that they belonged to a group somehow “better” than others, right-wing populist leaders in the United States, encouraged their followers to reject measures designed to reduce the spread of the COVID-19 infection, and many of their followers did so at significant risk and cost to their own health. For example, at the beginning of the pandemic President Trump downplayed the virus’s seriousness and promoted the idea that the supposed “exceptional” status of Americans would enable them to escape the virus’s devastation. Later, when the seriousness of the pandemic became more difficult to deny, Trump employed more overtly negative tactics, encouraging “real” Americans to reject public health mandates such as mask-wearing. As social identity theory would predict and as researchers have since demonstrated, many people acted according to these social identity cues. Studies show that Americans’ response to the pandemic was strongly influenced by their political identifications [10].

Right-wing populist conspiracy theories about the virus can also be interpreted in terms of social identity principles. Generally speaking, conspiracy theories appeal to individuals’ desire to see themselves and their groups as uniquely knowledgeable—and thus as “positively distinct” from others. The belief that one’s group has special, “secret” knowledge reinforces the illusion that group members are superior to those they view as less enlightened. The specific content of many right-wing populist conspiracy theories, however, went further. Many of them, such as the idea that the COVID-19 vaccination contains a computer chip designed by “the elite” to control “us” promoted social antagonisms, which in turn enhanced subjective feelings of group identification and ingroup superiority. Others gave their adherents permission to conceive of their enemies in wildly imaginative and/or ethnically compromising situations, which in turn allowed them to establish an even greater psychological distance from them. These theories also reinforced a sense of moral superiority by giving “the people” the chance to express horror over the imagined actions of “others.” The worse these others were said to be, the better members of the ingroup could feel about their group identification. Although group membership does not necessarily mean the acceptance of group opinions, party identification has been shown, at least in some cases, to be more decisive for belief formation than scientific literacy [11].

Trump also discouraged compliance with anti-COVID-19 public health measures by stirring up negative emotions, such as resentment, anger, and fear. Such emotional appeals are classic right-wing populist tactics, which also reflect social identity theory principles. Not only do they enhance the sense of superiority that ingroup members feel over outgroup members, but they also deepen the perceived difference between in and outgroup members. By fueling resentment against public health officials who sought to mitigate the severity of the pandemic, Trump gave his followers permission to revel in feelings of victimhood, thereby affirming their ‘special’ status. Similarly, by stoking resentment against public health regulations, he communicated to his followers that they were above the rules—and thus above “other,” rule-abiding citizens. Trump also aroused anger and fear when he blamed the virus on the Chinese. By proclaiming the Chinese dangerous and evil, he once again made it easy for his followers to imagine themselves as “better.” Likewise, he reinforced nationalistic sentiments by stoking fear about disease-carrying immigrants. This tactic gave his followers the opportunity to relish in a false sense of security, as it promoted the notion that “we” (“the pure people”) will not get sick if we keep “them” (immigrants and foreigners) out of the country. In each of these ways, Trump helped his followers achieve and preserve a positive self-image, allowing them to see themselves as belonging to a group that was both positively distinct from supposedly “contaminated” others and superior to public health elites. By exacerbating “us-them” distinctions and blaming outgroup members, Trump enhanced his followers’ sense of belonging and granted them a feeling of superiority at the same time.

Not surprisingly, these negative emotions and beliefs led to oppositional behavior. Right-wing populists who blamed the pandemic on policy elites and outsiders could easily deny their own responsibility to curb the virus’s spread. Moreover, many experienced a kind of illusionary freedom in their acts of defiance, especially when these were linked to group membership. By refusing to take virus-mitigating measures seriously, they experienced themselves as free simply because they distinguished themselves from the rule-following “sheep.” In line with what SIT would predict, the desire for this kind of experience, which affirms membership in a positively distinct, privileged social identity, may be more determinant of behavior than concern for one’s own health or the health of ingroup members. Perceived, imagined, or symbolically constructed advantages regularly trump real-life prospects, especially when dangers and risks appear abstract or distant.

## Part Three: Possible Solutions

One way in which this kind of negativity may be at least partially overcome is through an appeal to what social identity theorists call “superordinate identity”—the creation of a single, larger identity that encompasses conflicting subgroups. Originally proposed by social identity scholars who recognized the ease with which people may shift their salient identifications when primed to do so, highlighting a superordinate identity has been shown to reduce intergroup bias under certain circumstances [12]. Not surprisingly, then, top social identity theorists who addressed pandemic-related issues appealed to this idea, and public health messaging repeatedly implored people to work “together against the virus.” Unfortunately, however, this kind of messaging has not proven sufficient to overcome substantial elements of resistance.

Interestingly, social identity theory itself provides some clues as to why this is the case. When a group identity becomes too large, individuals often lose the sense of distinctiveness they seek [13]. It is difficult to feel special when one is forced to identify with all of humanity. Moreover, when everyone is included in a single superordinate group, the basis of comparison essential to self-understanding is lost. For this reason, it may be more productive to appeal to smaller superordinate identities, such as country citizenship. People may be more motivated to help their own society cope better than others than they are to support the more abstract notion of humanity’s victory. Of course, appealing to less expansive superordinate identities may come at the cost of increased antagonism toward outgroups. Ideally, public health campaigns would seek a dual focus, appealing both to the distinctiveness of specific groups and to humanirty as a universal group.

Another reason why appeals to a superordinate identity have not led to more compliance may be because the kind of compliance needed to defeat the virus requires long-term identification and commitment. Social identity theory’s experiments demonstrate the ease with which people’s salient group identification can shift when prompted, but they do not reveal much about the formation of long-term identifications. Thus, people who find themselves in immediate danger are often quick to set aside their differences and recognize their commonality [14]. However, in slow-burning crises like the pandemic and climate change, when the fire or flood is not yet at one’s door, the necessary shift in identification occurs slowly, if at all. In fact, knowledge of such looming, but apparently distant dangers has been shown to increase the probability that people will take sides against each other. For example, when people are reminded of the inevitability of their mortality (without being put in immediate danger), they become more concerned with establishing group distinctiveness [15].

Another problem is that some of the conditions known to promote the achievement of superordinate identity—such as the existence of groups with equal status and the opportunity for members from opposing groups to get to know each other—have been especially difficult to achieve during the pandemic. Not only has the pandemic severely reduced the opportunity for safe social interaction, but it has also exacerbated social inequalities. Lockdowns, for example, devastated restaurant and shop owners, but greatly benefitted large delivery companies like Amazon. The psychological costs and benefits of anti-COVID-19 measures also had varying implications, depending upon people’s unique circumstances. For example, teenagers and young adults, who have a high risk for mental health issues, suffered grave psychological costs during the lockdown with relatively little benefit. Although it would be impossible to take each individual’s costs and benefits into account, governments that take group variances into consideration are apt to produce better overall health outcomes. They will also be less likely to arouse resentment from those who feel they are bearing an unfair proportion of the burden of the mitigation measures.

Even with these refinements, the appeal to a superordinate identity is unlikely to be sufficient to quell resistance to vaccines and other public health measures designed to reduce the virus’s spread. What social identity theory teaches, above all, is the appeal of group identifications even when these undermine the welfare of one’s own group members. When right-wing populists build upon this social-psychological weakness and fan the flames of antagonistic identifications, international public health goals are easily undermined. To the extent that this resistance cannot be overcome through persuasion, mandates may be the best short-term solution.

Long-term approaches will also be essential. If this pandemic and other arising threats to global public health such as climate change are to be defeated, public health officials must acknowledge the radicalism of right-wing populism’s anti-health agenda and take political action against it. Physicians may be obliged to stay out of politics, but public health officials do not have this obligation (or luxury). We must lobby governments to legislate against misinformation campaigns, promote civic education, and implement programs that promote social equality and social cooperation among individuals with diverging political identifications. Perhaps most importantly, we must engage in a concerted effort to support individuals’ self-esteem through interpersonal recognition and mental health interventions so that people become less susceptible to the allure of group identifications that override rational concerns. Only with the implementation of such measures can a degree of public health be preserved.

## Data Availability

Publicly available datasets were analyzed in this study. This data can be found at references [7] and [16].
